# Intramedullary Spinal Cord Metastases Presenting As the First Manifestation of Lung Adenocarcinoma: Report of a Rare Case

**DOI:** 10.7759/cureus.95915

**Published:** 2025-11-01

**Authors:** Sarah Slimani, Sara El Ghaffouli, Youssouf Benmoh, Abderrahim Elktaibi, Ahmed Bourazza

**Affiliations:** 1 Department of Neurology, Mohammed V Military Teaching Hospital - Mohammed V University, Rabat, MAR; 2 Department of Pathology, Mohammed V Military Training Hospital, Faculty of Medicine and Pharmacy of Rabat, Rabat, MAR; 3 Department of Pathology, Ibn Sina University Hospital, Faculty of Medicine and Pharmacy of Rabat, Rabat, MAR; 4 Department of Neurology, Mohammed V Military Teaching Hospital, Rabat, MAR; 5 Department of Pathology, Mohammed V Military Teaching Hospital, Rabat, MAR; 6 Department of Neurology, Mohamed V Military Teaching Hospital, Mohamed V University, Rabat, MAR

**Keywords:** adenocarcinoma, intramedullary metastases, lung cancer, metastatic dissemination, mri

## Abstract

Intramedullary spinal cord metastases (ISCM) are a rare complication of cancer and rarely reveal it. We report a case of a 54-year-old man who experienced progressive weakness and numbness of his right leg, which caused him to have walking difficulties. The spinal cord MRI demonstrated centromedullary high signal intensity in the cervicodorsal region, suggesting secondary locations. The patient underwent a CT scan of the chest, abdomen, and pelvis, which showed a lung tumor. The lung biopsy revealed an acinar-predominant adenocarcinoma. The patient's treatment involved both chemotherapy and radiation therapy, and he died two months after the diagnosis.

## Introduction

Intramedullary spinal cord metastases (ISCM) are extremely rare complications of cancers: they account for 3.4 to 5% of spinal cord disorders in cancer patients and represent 1 to 3% of all intramedullary tumors. They are most commonly found in lung cancer (>50% of cases) and also in breast cancer, melanoma, blood cancer, renal cell carcinoma, and colorectal cancer [1-3].

The metastatic dissemination is arterial, which may explain its deep location in the spinal cord, near the territory of the spinal arteries. Other proposed mechanisms include retrograde infiltration via the spinal cord's venous system and perineural invasion, also known as neurotropic malignancy.

Exceptionally, symptoms from ISCM may also be the first presentation of an unknown primary cancer. Most patients present with progressive sensory-motor deficits, pain, or urinary incontinence [1,4,5]. These nonspecific symptoms may lead to a delay in the diagnosis as a result. MRI with and without contrast is the main diagnostic tool for spinal cord metastatic disease. Generally, there is a single lesion in the thoracic cord [3]. Early diagnosis is imperative for improved treatment outcomes, which can prevent serious sensory-motor deficits and sphincter dysfunction. In this report, we describe a rare case of a lung adenocarcinoma ISCM, aiming to highlight the importance of recognizing cancer early in its atypical presentations.

## Case presentation

A 54-year-old man with a history of cataract presented with progressive worsening weakness and numbness of his right leg for two months, which caused him difficulty walking without bowel or bladder changes.

**Figure 1:**
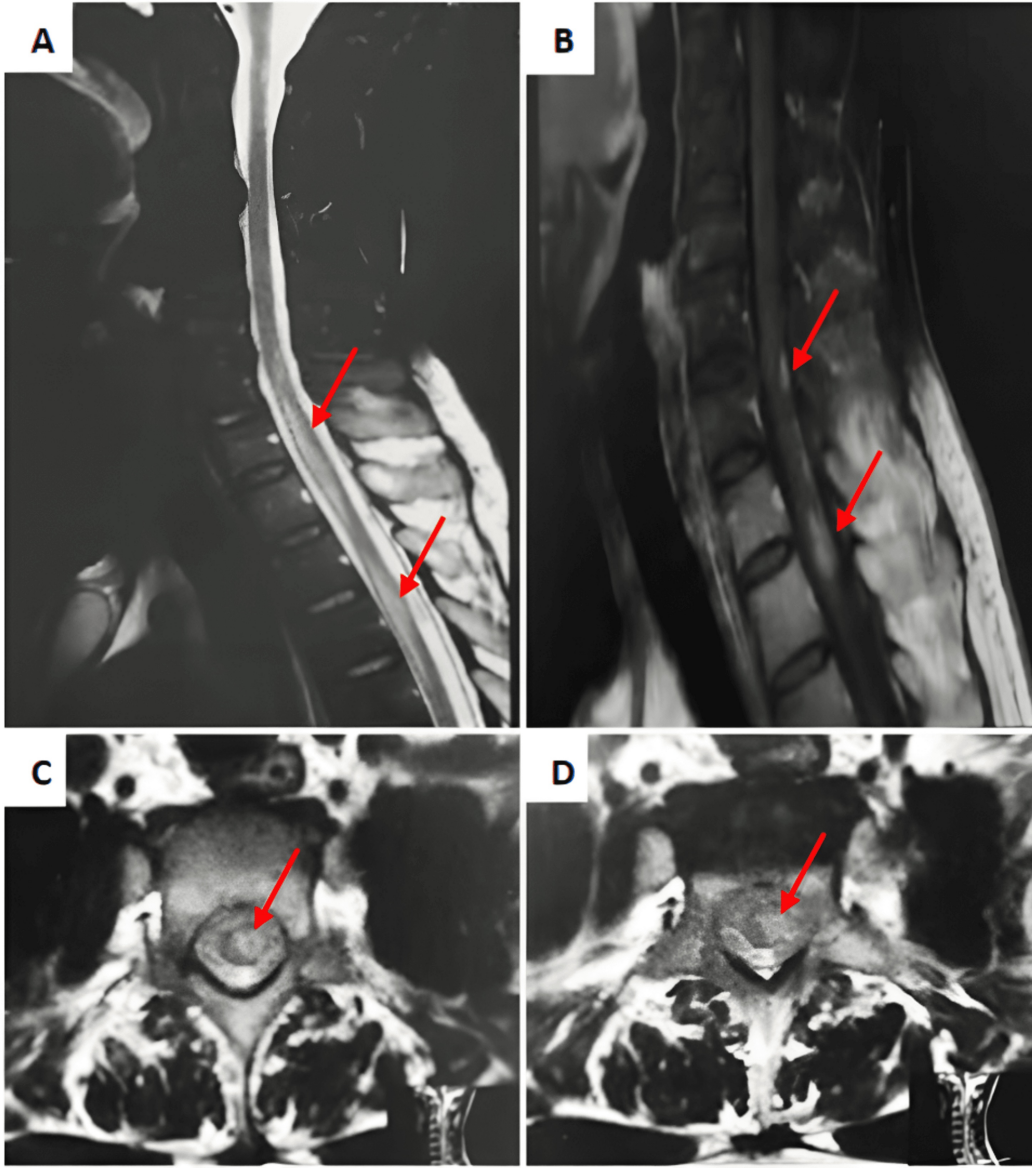
MRI of the cervical and thoracic spine demonstrating intramedullary spinal cord metastases (red arrows in images A, B, C, and D). (A) Sagittal T2-weighted MRI showing a hyperintense lesion with surrounding edema. (B) Sagittal post-contrast T1-weighted MRI showing a peripheral thin region of increased enhancement (rim sign) associated with an ill-defined enhancement extending below the lesion. (C-D) Axial T2-weighted MRI.

Neurological examination revealed 4/5 strength in the right lower limb without muscle atrophy, hyperreflexia of the upper and lower limbs with right positive Babinski’s sign, and decreased sensation to light touch, pinprick, and temperature stimuli in bilateral lower extremities below the level of T-10 dermatome. His symptoms were evaluated with magnetic resonance imaging (MRI) of the spine with and without contrast. The MRI demonstrated centromedullary high signal intensity in the cervicodorsal region, showing enhancement, suggesting secondary locations (Figure 1).

The patient underwent a CT scan of the chest, abdomen, and pelvis to identify the primary neoplasm. The scan showed a lung tumor in the dorsal segment of the right upper lobe with mediastinal lymphadenopathy, left adrenal, and liver metastases.

**Figure 2:**
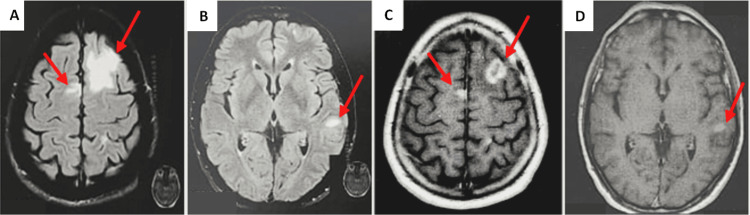
Axial fluid-attenuated inversion recovery-weighted (FLAIR) and post-contrast T1-weighted MRI of the brain demonstrating brain metastases. (A) Left anterior (right red arrow in image A) and right parasagittal (left red arrow in image A) frontal metastases, annular in appearance after contrast-enhancement (red arrows in image C) surrounded by a very large amount of finger glove edema clearly visible on FLAIR imaging (A) associated to an enhancing focus involving the left parietal lobe (red arrow in image B and D), consistent with intracranial metastatic disease.

A brain MRI was also performed and showed multiple intraparenchymal lesions, hyperintense on fluid-attenuated inversion recovery-weighted (FLAIR) and on T2-weighted images, with nodular or annular enhancement surrounded by vasogenic edema consistent with intracranial metastatic disease (Figure 2).

**Figure 3:**
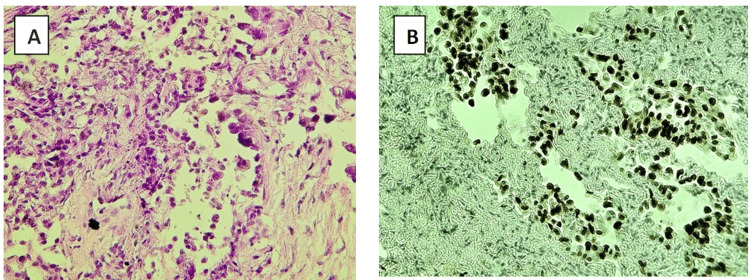
Histological evaluation of the lung biopsy. (A) H&E staining (original magnification ×20) showing tumor cells. (B) Positive thyroid transcription factor-1 staining, indicating the lung origin.

Pathologic examination of the lung biopsy revealed an acinar-predominant adenocarcinoma (Figure 3). The tumor was immunologically positive for thyroid transcription factor-1 as well as negative for p63.

The patient received whole-brain and spine radiation therapy combined with chemotherapy. He died within one month of the start of chemotherapy.

## Discussion

Spinal cord metastases are extremely rare, occurring in 0.1 to 0.4% of patients with cancer [6]. They account for 1% of all spinal tumors and 1-3% of intramedullary tumors [7,8]. ISCM are rarely isolated and are often associated with other metastases, particularly cerebral ones. Lung cancer is the most common primary tumor (48% of cases), followed by breast cancer (16% of cases) [9]. These two primary tumors are also the most frequent causes of cerebral metastases, with 40% for lung cancer and 19% for breast cancer. This might be partly explained by the fact that they have the highest incidence in a large population.

The pathophysiology of cord infiltration by a metastatic tumor remains unclear. It might be the result of hematogenous dissemination, which leads to arterial embolization or involves retrograde infiltration via the spinal cord venous system (Batson plexus) [3].

The distribution of ISCM primarily depends on the length of the affected segment. In most studies, dorsal ISCM are more frequent, followed by cervical and lumbar ISCM; multiple locations aren’t uncommon [10]. In the case of our patient, the metastases were multiple (three) and located in the cervical and dorsal regions. The symptomatology of ISCM may be the initial presentation of the cancer, but in most cases, it appears after the diagnosis of the primary tumor. Generally, ISCM cause edema, distortion, and compression of the spinal cord parenchyma, leading to pain, sensory disturbances, followed by weakness, and eventually bladder and bowel dysfunction [11]. Asymptomatic presentation isn’t uncommon [12]. Unlike the few reported cases, our patient had no neuropathic pain, bowel, or bladder changes. The onset of his symptoms and the worsening weakness lasted two months. As it's known, the rapid worsening of neurological symptoms within less than a month distinguishes ISCM from primary intramedullary tumors, whose progression is generally slower [8].

MRI is by far the most sensitive imaging modality recommended for the detection of ISCM. The examination should be as thorough as possible, including the entire spinal axis. For the tumor region, at least two orthogonal planes should be performed, most often with sagittal and axial T2-weighted and pre- and post-contrast T1-weighted sequences, as well as diffusion tensor imaging. A supplementary brain imaging is also recommended [13,14].

ISCM have a nonspecific MRI presentation, with cord swelling, edema, and an enhancing lesion [3]. Recently described “rim” and “flame” signs help to differentiate intramedullary metastases from primary spinal cord tumors. The first sign is characterized by Rykken and colleagues as a total or partial rim of gadolinium enhancement, and the second sign is explained as a blurry, flame-shaped, gadolinium-enhancing region at the superior or inferior border of a well-defined lesion [3].

Other differential diagnoses reported in the literature for imaging of ISCM, in addition to primary spinal cord tumor, include hemangioblastoma, demyelinating diseases of the central nervous system, arteriovenous malformations, sarcoidosis, and transverse myelitis [15].

The large range of clinical situations and the dearth of controlled trials on the effects of various treatment choices make it challenging to manage patients with ISCM optimally. Treatment options for ISCM are often palliative and include chemotherapy, radiotherapy ( mostly stereotactic radiotherapy at the moment), microsurgical excision, and palliative treatment, particularly steroids. Radiation treatment has been recommended by several authors, mostly for radiosensitive metastases such as small cell carcinoma, breast cancer, or lymphoma [4,8,16]. It has been shown to have no known toxicity and to produce satisfactory local control and clinical improvement [16].

Treatment choices should involve analysis of possible benefits (that are, pain relief, ambulation, alleviation of incontinence) over the course of the patient’s life expectancy.

The treatment in this case was based on palliative options and did not include surgery, given the stage of the disease, the number of secondary lesions, and the absence of surgical indications for brain metastases. A subsequent reassessment was planned.

Among patients who undergo surgery (for radioresistant solitary metastases), 58% experience functional improvement, while 11% show deterioration. In contrast, of those managed conservatively, 21% improve, 63% remain stable, and 17% experience a decline in function [2].

The prognosis for pulmonary-origin ISCM remains poor, with a median survival of six months for patients who undergo surgery, five months for those treated conservatively, and only one month for those receiving palliative care [2,9]. Our patient died within one month.

## Conclusions

The rapid progression, multifocal involvement, and the fact that ISCM was the first sign of malignancy make reporting such a rare case valuable, raising awareness for early recognition of cancer in atypical presentations.

Due to the poor prognosis of this condition, the discovery in the years to come of new therapies such as immunotherapy, targeted and gene therapy, and minimally invasive ablative techniques could improve the course of a patient’s life expectancy.

## References

[1] Goyal A, Yolcu Y, Kerezoudis P, Alvi MA, Krauss WE, Bydon M (2019). Intramedullary spinal cord metastases: An institutional review of survival and outcomes. J Neurooncol.

[2] Hata Y, Takai Y, Takahashi H (2013). Complete response of 7 years’ duration after chemoradiotherapy followed by gefitinib in a patient with intramedullary spinal cord metastasis from lung adenocarcinoma. Journal of Thoracic Disease.

[3] Merhemic Z, Stosic-Opincal T, Thurnher MM (2016). Neuroimaging of spinal tumors. Magn Reson Imag Clin N Am.

[4] Majmundar N, Shao B, Assina R (2018). Lung adenocarcinoma presenting as intramedullary spinal cord metastasis: Case report and review of literature. J Clin Neurosci.

[5] Kalaycı M, Çağavi F, Gül S, Yenidünya S, Açıkgöz B (2004). Intramedullary spinal cord metastases: Diagnosis and treatment - an illustrated review. Acta Neurochir.

[6] Gasser TG, Pospiech J, Stolke D, Schwechheimer K (2001). Spinal intramedullary metastases. Report of two cases and review of the literature. Neurosurg Rev.

[7] Sung W-S, Sung M-J, Chan JH (2013). Intramedullary spinal cord metastases: A 20-year institutional experience with a comprehensive literature review. World Neurosurg.

[8] Dam-Hieu P, Seizeur R, Mineo J-F, Metges J-P, Meriot P, Simon H (2009). Retrospective study of 19 patients with intramedullary spinal cord metastasis. Clin Neurol Neurosurg.

[9] Ding D, Fullard M, Jarrell HS, Jones DE (2014). Intramedullary spinal cord metastasis from salivary ductal carcinoma of the parotid gland mimicking transverse myelitis in a patient with radiologically isolated syndrome. Journal of the Neurological Sciences.

[10] Potti A, Abdel-Raheem M, Levitt R, Schell DA, Mehdi SA (2001). Intramedullary spinal cord metastases (ISCM) and non-small cell lung carcinoma (NSCLC): Clinical patterns, diagnosis and therapeutic considerations. Lung Cancer.

[11] Isla A, Paz JM, Sansivirini F, Zamora P, García Grande A, Fernandez A (2000). Intramedullary spinal cord metastasis. A case report. J Neurosurg Sci.

[12] Grasso G, Meli F, Patti R, Giambartino F, Florena AM, Iacopino DG (2007). Intramedullary spinal cord tumor presenting as the initial manifestation of metastatic colon cancer: Case report and review of the literature. Spinal Cord.

[13] Cosnard G, Duprez T, Grandin C, Hernalsteen D (2010). Tumor and pseudo-tumoral spinal cord. (Article in French). J Radiol.

[14] Cosnard G (2012). Pitfalls and tips in spinal cord pathology. (Article in French). Journal de Radiologie Diagnostique et Interventionnelle.

[15] Do-Dai DD, Brooks MK, Goldkamp A, Erbay S, Bhadelia RA (2010). Magnetic resonance imaging of intramedullary spinal cord lesions: A pictorial review. Curr Prob Diagnos Radiol.

[16] Tonneau M, Mouttet-Audouard R, Le Tinier F, Mirabel X, Pasquier D (2021). Stereotactic body radiotherapy for intramedullary metastases: A retrospective series at the Oscar Lambret center and a systematic review. BMC Cancer.

